# Satellite Towns

**Published:** 1924-02

**Authors:** 


### "SATELLITE TOWNS."

COBBETT was wont to describe London as "the great wen," yet in his day London was no more than a microcosm of its present self. It has been unpleasantly obvious for some considerable time that it is now too large for comfort, and the recent astonishingly rapid increase in motor traffic has made the problem of the streets so desperate that a special police official has had to be appointed to deal with it, and Parliament is about to be asked to take a hand in the game. But neither police nor Parliament can arrest the growth of the capital—we cannot issue permits to visit or reside in it, and in ten years' time the congestion will probably be much greater than now. How, then, are we to solve this apparently insoluble problem ? Mitigate it we must if we are ever to get rid of overcrowding and slums. The difficulty is not confined to London. Great towns are suffering in much the same way, and small towns are rapidly growing great, and developing identical difficulties. The idea of the "satellite town" as, at all events, a partial remedy is not new, but it has just been brought into prominence in a lecture on the subject by Sir Theodore Chambers, the Chairman of the Welwyn Garden City.

The remedy offered by the Garden City, or "Satellite Town," is to cut slices out of London and plant them down elsewhere. There are great numbers of industries, at present centred in the capital and other great towns, which could equally well, and much more cheaply, be conducted in the country, where sites are cheaper, rates lower, and fresh air abundant. The removal of those who are dependent upon these industries would make it comparatively easy to clear those slums which we are at present compelled to keep in being because of the necessity of rehousing the dispossessed. Much of the death-rate, and still more of the sickness-rate, of the towns is due to overcrowded areas which depress the general average of health. The creation of fresh suburbs is no remedy at all—the greater the number of "dormitories" the worse the central congestion. Within the 400 square miles of what we call "London" there is a population of six millions and three quarters, or thirty persons to the acre. Yet within the Home Counties there is an equal area with a population of no more than .3 per acre.

We can hardly look to the State for the creation of these towns. Like Letchworth and Welwyn, we shall owe them, when we get them, to private enterprise in which enlightened public interest will go hand in hand with business acumen. At the worst, and even if they failed noticeably to reduce the congestion of London, these towns would do much to prevent its further growth. They are, or should be, sufficiently distant and sufficiently self-contained to present little attraction to the day-worker in the capital. It is, in fact, in their self-contained character that much of their value lies. They are new towns with a conscious corporate existence, endowed with all the essentials, in a saner form, of all other towns, but armed with experience and facilities for avoiding the difficulties and dangers which assail the old urban settlements that have grown up haphazard.

